# Diagnostic value and prognostic implications of serum procalcitonin after cardiac surgery: a systematic review of the literature

**DOI:** 10.1186/cc5067

**Published:** 2006-10-13

**Authors:** Christoph Sponholz, Yasser Sakr, Konrad Reinhart, Frank Brunkhorst

**Affiliations:** 1 Department of Anesthesiology and Intensive Care, Friedrich-Schiller-University, Erlanger Allee 103, 07743 Jena, Germany

## Abstract

**Introduction:**

Systemic inflammatory response syndrome is common after surgery, and it can be difficult to discriminate between infection and inflammation. We performed a review of the literature with the aims of describing the evolution of serum procalcitonin (PCT) levels after uncomplicated cardiac surgery, characterising the role of PCT as a tool in discriminating infection, identifying the relation between PCT, organ failure, and severity of sepsis syndromes, and assessing the possible role of PCT in detection of postoperative complications and mortality.

**Methods:**

We performed a search on MEDLINE using the keyword 'procalcitonin' crossed with 'cardiac surgery,' 'heart,' 'postoperative,' and 'transplantation.' Our search was limited to human studies published between January 1990 and June 2006.

**Results:**

Uncomplicated cardiac surgery induces a postoperative increase in serum PCT levels. Peak PCT levels are reached within 24 hours postoperatively and return to normal levels within the first week. This increase seems to be dependent on the surgical procedure and on intraoperative events. Although PCT values reported in infected patients are generally higher than in non-infected patients after cardiac surgery, the cutoff point for discriminating infection ranges from 1 to 5 ng/ml, and the dynamics of PCT levels over time may be more important than absolute values. PCT is superior to C-reactive protein in discriminating infections in this setting. PCT levels are higher with increased severity of sepsis and the presence of organ dysfunction/failure and in patients with a poor outcome or in those who develop postoperative complications. PCT levels typically remain unchanged after acute rejection but increase markedly after bacterial and fungal infections. Systemic infections are associated with greater PCT elevation than is local infection. Viral infections are difficult to identify based on PCT measurements.

**Conclusion:**

The dynamics of PCT levels, rather than absolute values, could be important in identifying patients with infectious complications after cardiac surgery. PCT is useful in differentiating acute graft rejection after heart and/or lung transplantation from bacterial and fungal infections. Further studies are needed to define cutoff points and to incorporate PCT levels in useful prediction models.

## Introduction

Procalcitonin (PCT) is a polypeptide consisting of 116 amino acids and is the precursor of calcitonin [1]. The role of PCT in inflammatory conditions, such as sepsis, was first described by Assicot *et al*. [2], who observed a rise in serum PCT levels three to four hours after a single injection of endotoxin, reaching a maximum 24 hours thereafter [3]. The origin of PCT in the inflammatory response is not yet fully understood, but it is believed that PCT is produced in the liver [4] and peripheral mononuclear cells [5], modulated by cytokines and lipopolysaccharide.

Over the last decade, PCT has become increasingly popular as a novel marker of infection in the intensive care unit (ICU) setting. Several studies have underscored its value in a variety of clinical conditions for identifying infectious processes [6-8], characterising the severity of the underlying illness [9,10], guiding therapy [11-13], and risk stratification [14-16]. Three different kits for PCT measurement are currently available: the LUMI-Test, the Q-Test, and the Kryptor Test (BRAHMS AG, Hennigsdorf, Germany). The most commonly used kit for measuring PCT, the LUMI-Test, is based on a immunoluminometric assay that binds PCT to two different antibodies in the calcitonin and katacalcin regions of the protein. Results of the measurement are available within one hour, and only 20 μl of blood serum or plasma is needed for the test. The sensitivity of the LUMI-Test is 0.1 ng/ml [17], the functional assay sensitivity (defined as the smallest value with an interassay precision of 20% coefficient of variation [CV]) is 0.3 ng/ml, and the interassay precision in the clinically relevant range is between 6% and 10% CV (data provided by BRAHMS AG). The PCT Q-Test uses a semiquantitative one-step, solid-phase immunoassay that needs 200 μl of serum or plasma, with results available within 30 minutes. The semiquantitative measurement of the test is correlated to three reference concentrations of 0.5, 2, and 10 ng/ml [18,19]. The Kryptor Test for PCT measurement was introduced in 2004. This test is based on TRACE (time-resolved amplified cryptate emission) technology. PCT values are available within 20 minutes with a functional assay sensitivity of 0.06 ng/ml, and 50 μl of blood serum or plasma is needed for measurement [20]. At a concentration between 0.1 and 0.3 ng/ml, the Kryptor Test has an intra-assay CV of less than or equal to 7% and an interassay CV of less than or equal to 10%, and at concentrations greater than 0.3 ng/ml the intra-assay CV is less than or equal to 3% and the interassay CV is less than or equal to 6% (data provided by BRAHMS AG).

Surgical patients, especially those admitted to the ICU after cardiac surgery, represent a major diagnostic challenge in terms of identification of infectious complications. These patients have usually been subjected to intraoperative procedures that may induce various degrees of tissue inflammation and cytokine liberation [21]. Meisner *et al*. [22] reported that PCT concentrations were moderately increased above the normal range in 32% of patients after minor and aseptic surgery, in 59% after cardiac and thoracic surgery, and in 95% of patients after surgery of the intestine. Cardiac surgery *per se *and the use of cardiopulmonary bypass (CPB) lead to a more pronounced activation of cytokines than that following some other surgical procedures [23]. This cytokine 'burst' leads to a systemic response by the body's inflammatory system, well known as the systemic inflammatory response syndrome (SIRS) [24] and similar to that observed with infections, making the diagnosis of infection more difficult. Because timing is crucial in initiating therapy and determining the subsequent outcome of septic conditions [25], understanding the kinetics of PCT in various clinical conditions may improve our ability to use this marker as an early diagnostic tool.

The aims of this qualitative review were, therefore, to identify the time course of serum PCT levels after uncomplicated cardiac surgery, to characterise the possible differences in serum PCT levels with various surgical procedures, and to investigate the value of PCT levels in terms of diagnosing infection or predicting outcome in these patients.

## Materials and methods

We performed a search on MEDLINE using the keyword 'procalcitonin' crossed with 'cardiac surgery,' 'heart,' 'postoperative,' and 'transplantation.' Our search was limited to human studies published between January 1990 and June 2006. The abstracts of all articles were used to confirm our target population (patients undergoing cardiac surgery), and the corresponding full-text articles were reviewed for the presence of data with postoperative PCT levels. Two investigators (CS and YS) independently identified the eligible literature. Among the pre-defined variables collected were year of publication, study design (prospective/retrospective/case report), number of patients included, age group (adults or infants), disease group, markers other than PCT, and study results. Any inconsistencies between the two investigators in the data collected were resolved by consensus. To avoid publication bias, abstracts and full articles were eligible if PCT levels were reported. We also reviewed the bibliographies of available studies for potentially eligible studies. Of 37 articles that quoted PCT levels in patients undergoing cardiac surgery, three articles in abstract form were excluded because of insufficient data [26-28] and 34 were included in our review. Table 1 gives an overview of the studies included.

### Time course of serum PCT levels after uncomplicated cardiac surgery

**Figure 1:**
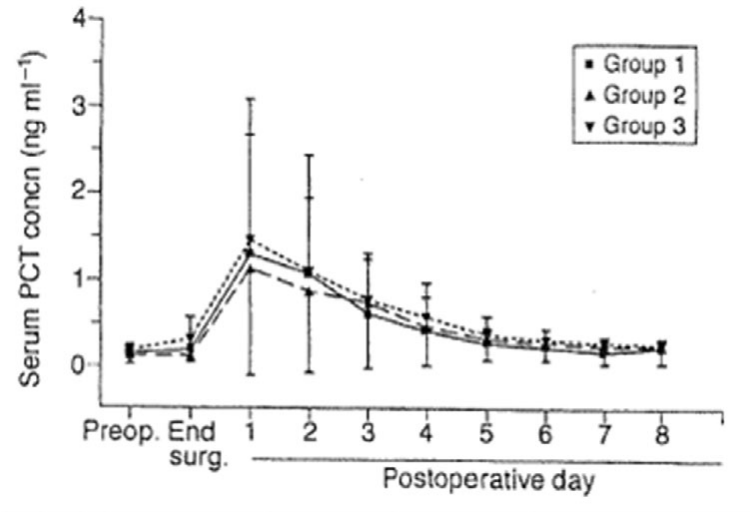
Serum procalcitonin (PCT) concentrations in patients after cardiac surgery with no complications according to the type of surgery. Group 1, coronary artery bypass grafting (CABG) with cardiopulmonary bypass (CPB). Group 2, CABG without CPB. Group 3, valvular surgery with CBP.^© ^The Board of Management and Trustees of the British Journal of Anaesthesia. Reproduced from [36] by permission of Oxford University Press/British Journal of Anaesthesia.

Serum PCT levels increase postoperatively after uncomplicated cardiac surgery, reaching a peak level within 24 hours postoperatively [29-48], and return to normal values in the following days (Figure 1). Peak PCT values, measured by the immunoluminometric assay, range from 0.5 to 7.0 ng/ml [29,31-40,42-44,46-48].

Several factors may influence the evolution of serum PCT levels after cardiac surgery in the absence of postoperative complications. The specific surgical techniques used during the procedure may be one important factor. Franke *et al*. [34] reported higher PCT levels in patients after on-pump coronary artery bypass grafting (CABG) than in those after off-pump coronary artery bypass (OPCAB) surgery. Kilger *et al*. [29] found higher postoperative PCT levels in patients after OPCAB than in those after minimally invasive direct coronary artery bypass (MIDCAB), with median PCT levels of 2.0 ng/ml in the OPCAB and 0.7 ng/ml in the MIDCAB group. PCT levels were also higher after valvular surgery and thoracic aortic surgery than after CABG, with Loebe *et al*. [40] reporting PCT levels of greater than or equal to 5 ng/ml in 13% of patients who underwent CABG compared with 39% and 35% of those who underwent valvular and aortic surgery, respectively. A more pronounced increase in serum PCT levels was also reported after procedures involving ventriculotomy than after those involving atriotomy [48,49]. In paediatric patients, PCT levels increased more markedly after surgical repair of Tetralogy of Fallot than in those undergoing repair of ventricular septal defect or atrial septal defect [50]. Intraoperative factors have also been shown to influence the postoperative evolution of serum PCT levels (for example, aortic cross-clamping time [30,46,48,49], duration of CPB [30,45,46], and the duration of surgery [45]).

Elevated PCT levels after surgical procedures may be explained by normal PCT kinetics. Three to four hours after injection of endotoxin in healthy subjects, PCT levels start to rise, reaching a maximum 24 hours thereafter [3]. The return in PCT levels to normal within a few days after surgery after an uncomplicated postoperative course can be explained by the half-life of PCT (18 to 24 hours) [1] in the absence of a further insult that may induce more PCT production. Meisner *et al*. [51] showed that PCT production could be induced by various stimuli such as trauma, tissue injury, and others and that this non-specific and non-infectious stimulation of PCT is much lower than specific induction and much lower compared with other markers of the inflammatory response. The source of PCT production in these conditions could be explained by non-specific cytokine liberation from the injured tissue [52]. Endotoxin release has also been reported after procedures involving the heart-lung machine [53].

The evolution of other clinically used markers of tissue inflammation/infection in relation to that of PCT was also reported in some comparative studies. C-reactive protein (CRP) levels increase postoperatively, peaking between postoperative days one and three and remaining elevated up to the second week postoperatively [29,30,35,54]. irrespective of the extent of surgery [33,34,36,38,42,45,46,54]. Levels of interleukin-6 (IL-6), another marker of immune system activation, also increase postoperatively [34,41,46,48,50,55,56]., peaking at 6 to 24 hours after surgery [34,46,48], and are probably not related to the extent of surgery [48].

In summary, uncomplicated cardiac surgery induces a postoperative increase in serum PCT levels. Peak PCT levels are reached within 24 hours postoperatively and return to normal levels within the first week. This increase seems to be dependent on the surgical procedure, with more invasive procedures associated with higher PCT levels, and on intraoperative events, including aortic cross-clamping time, duration of CPB, and the duration of surgery.

### PCT as a tool for identifying infection

Because of the marked overlap of signs and symptoms, diagnosis of infection still represents a major challenge in ICU patients after cardiac surgery. Early differentiation between SIRS after cardiac surgery and the development of perioperative infection is crucial to enable appropriate antibiotic therapy to be started and to prevent subsequent complications.

Several studies reported higher PCT levels after cardiac surgery in infected compared with non-infected patients [33,35-38,41,45,57,58]. Importantly, PCT levels remained elevated in the first week postoperatively [35,37,38]. The elevations in PCT levels were also reported to be more pronounced in bacterial and fungal infections than in viral infections of SIRS [37,59]. PCT levels ranged from a mean value of 4 ng/ml up to 30 ng/ml in infected patients, depending on the time at which infection was diagnosed. Initiating appropriate antibiotic therapy seems to bring about a marked reduction in PCT levels. Rothenburger *et al*. [35] reported a decrease in PCT levels in patients with systemic infection after cardiac surgery within 5 days after starting appropriate antibiotic therapy (from a median of 11 to 0.56 ng/ml).

In addition to PCT, CRP levels increase consistently after infection [35,38,40] and both seem to be correlated to infection in this subgroup of patients [38]. In contrast to PCT and CRP, white blood cell (WBC) count has no discriminative power in differentiating infected from non-infected patients after cardiac surgery [33,38,54].

**Figure 2:**
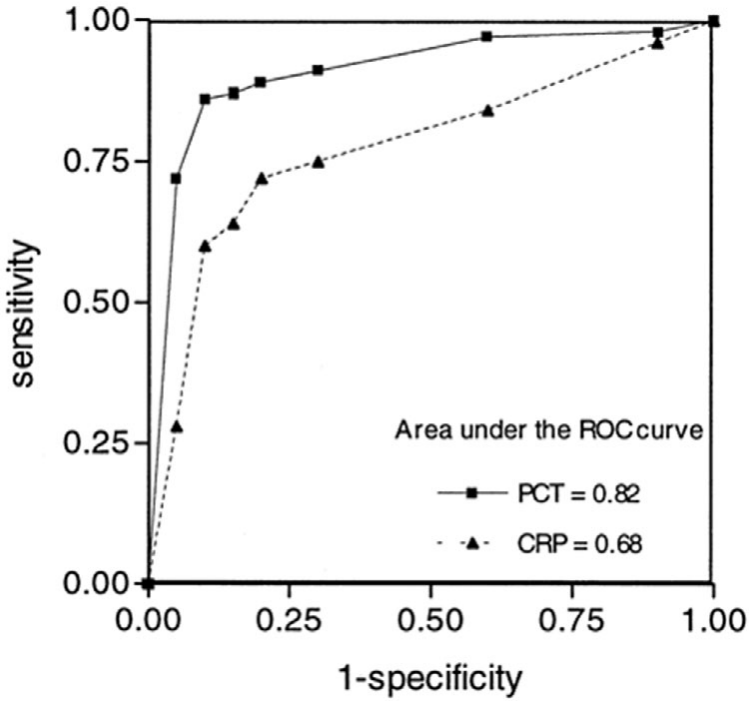
Procalcitonin (PCT) or C-reactive protein (CRP) to predict infection. Receiver operating characteristic (ROC) curve for PCT and CRP values for prediction of infection. From [39] with permission.

Rothenburger *et al*. [35] evaluated the diagnostic value of PCT and CRP in a group of 59 patients undergoing CPB. At a cutoff level of 4 ng/ml, PCT had a sensitivity of 86% and a specificity of 98% in predicting infection, whereas CRP at a cutoff level of 180 mg/l had a sensitivity of 100% and a specificity of 75%. Likewise, Aouifi *et al*. [39] reported that PCT was superior to CRP in predicting an infectious aetiology in 131 adult patients undergoing CPB. At a 1 ng/ml cutoff point, PCT had a sensitivity of 85% and a specificity of 95% in predicting infection, whereas CRP had a sensitivity of only 64% and a specificity of 84% at a cutoff level of 150 mg/l. Moreover, the area under the receiver operating characteristic (ROC) curves for prediction of infection was 0.82 and 0.62 for PCT and CRP, respectively (Figure 2). In addition, in 80 high-risk patients with an APACHE (acute physiology and chronic health evaluation) II score of greater than 20 undergoing CABG, Dörge *et al*. [57] found that PCT levels greater than 5 ng/ml had a sensitivity of 82% for discrimination of an infectious process but a poor specificity (only 45%). Meisner *et al*. [45] investigated the diagnostic value of PCT in predicting microbiologically proven infection in patients undergoing elective cardiovascular surgery. PCT levels greater than 2 ng/ml had a sensitivity of 83% and a specificity of 75% in this respect.

In summary, PCT values reported in infected patients are generally higher than in non-infected patients after cardiac surgery. PCT is superior to CRP in discriminating infections in this setting. PCT levels decrease markedly after initiation of appropriate antibiotic therapy. The dynamics of PCT levels, rather than the absolute values, could be important in identifying patients with infectious complications after cardiac surgery.

### The relation between PCT, organ failure, and severity of sepsis syndromes

Several studies have suggested the presence of a correlation between serum PCT levels, the severity of sepsis syndromes, and the occurrence of organ dysfunction/failure after cardiac surgery [36,37,41,44,59]. Aouifi *et al*. [36] reported that PCT levels were correlated with the severity of sepsis. PCT levels reached up to 20 ng/ml in patients with sepsis and were as high as 97 ng/ml in patients who developed septic shock after CBP. Sablotzki *et al*. [41,55] reported an elevation in PCT levels of more than 20 ng/ml during the first 3 days in patients suffering from multiorgan dysfunction syndrome (MODS) compared with patients with SIRS. Boeken *et al*. [59] reported mean PCT levels of 19 ng/ml in patients with sepsis, whereas sepsis-free patients had a mean PCT value of only 0.8 ng/ml. Recently, Celebi *et al*. [30] reported that PCT levels greater than 0.7 ng/ml, using the Kryptor assay, could predict postoperative organ failure in children undergoing cardiac surgery with a sensitivity of 85% and a specificity of 58%; at a cutoff level of 7.7 ng/ml, sensitivity and specificity rose to 100%. Brunkhorst *et al*. [9] found that PCT levels greater than 2 ng/ml discriminated patients with severe sepsis but not those with septic shock.

From the available literature, it is difficult to recommend cutoff points for discriminating patients according to the presence of organ dysfunction/failure or the severity of sepsis syndromes after cardiac surgery. In a group of 101 critically ill patients, Giamarellos-Bourboulis *et al*. [60,61] failed to demonstrate any agreement between standard definitions of sepsis syndromes and those incorporating PCT levels as part of the diagnostic criteria.

Comparative data with other markers of tissue inflammation are scanty. Only two studies reported higher IL-6 levels, equivalent to the increase in PCT, in patients developing MODS on the first postoperative day compared with patients with SIRS without evidence of organ failure [41,55], and WBC count [44] was poorly discriminative in this respect [41,55].

In summary, PCT levels are higher with increased severity of sepsis syndromes and the presence of organ dysfunction/failure. Interpretation of PCT levels in this context should take these factors into consideration. PCT levels are correlated to the severity of sepsis syndromes; however, it is difficult to recommend cutoff points from the current literature.

### The role of PCT in predicting postoperative complications and death

The association between serum PCT levels and the severity of sepsis syndromes and organ dysfunction/failure has created interest in the possible prognostic value of PCT levels. PCT levels have been shown to be correlated to several severity-of-illness scoring systems used in clinical practice, including APACHE II [45] and SAPS (simplified acute physiology score) II [39,45] scores. In addition, PCT levels correlated well with the degree of organ dysfunction/failure as assessed by the SOFA (sequential organ failure assessment) score [45]. Meisner *et al*. [45] showed that PCT levels correlated well to the maximum values of SOFA score over the first 2 postoperative days in 208 patients undergoing CPB. Indeed, several studies [40,41,55,57,62] have reported higher PCT levels in non-survivors after cardiac surgery compared with survivors. However, the discriminative power of PCT in this respect has been less investigated [41,55,57,62]. Dörge *et al*. [57] found that PCT levels greater than 10 ng/ml 24 hours postoperatively could discriminate non-survivors in a high-risk group of patients after CPB with a sensitivity of 72% but with a low specificity (51.3%). However, Fritz *et al*. [62] reported that a PCT level as low as 2.8 ng/ml was the best cutoff for predicting 28-day mortality in patients after CABG. Similarly, Celebi *et al*. [30] reported predictive values of postoperative PCT for mortality in children undergoing cardiac surgery. At a cutoff level of 34.2 ng/ml, PCT had a sensitivity of 100% and a specificity of 90%, whereas at a cutoff level of 5 ng/ml, PCT had a sensitivity of 100% and a specificity of 65% in predicting mortality.

PCT levels were also found to be related to the development of postoperative complications [42,45,46,57,63]. Lecharny *et al*. [42] described higher mean PCT levels in patients who developed postoperative myocardial infarction than in those with an uneventful postoperative course. Meisner *et al*. [45] demonstrated a correlation between postoperative PCT levels in terms of the development of SIRS, respiratory failure, and the need for positive inotropic support. Likewise, Dörge *et al*. [57] reported higher PCT levels in patients who developed postoperative organ failure than in those with an uncomplicated postoperative course. Adamik *et al*. [63] reported that after CPB, PCT levels remained unchanged in patients with an uneventful recovery and increased in patients with complications, especially in those who developed renal and hepatic dysfunction in addition to respiratory and circulatory insufficiency. Using a cutoff value of just 2.0 ng/ml, the positive and negative predictive values for postoperative complications were 100%/93% and 100%/87% on the first and second postoperative days, respectively. CRP does not seem to be useful as a prognostic marker [36], likely due to its prolonged elevation after an uneventful postoperative course.

In summary, PCT levels are consistently higher in patients with a poor outcome and in those who develop postoperative complications. Further studies are needed to define cutoff points and to incorporate PCT levels in useful prediction models.

### Role of PCT in monitoring patients after heart transplantation

Another potentially useful implication of serum PCT measurement is the differential diagnosis of postoperative complications in critically ill patients who have undergone heart transplantation. Differentiation between postoperative infection and rejection is important in order to be able to initiate appropriate therapy. Several studies [37,58,64-66] have evaluated the role of PCT in patients after heart and/or lung transplantation. In 12 patients undergoing endomyocardial biopsy after heart transplantation, Boeken *et al*. [37] described elevated PCT levels in patients with proven bacterial or fungal infection, whereas patients who developed graft rejection had almost normal PCT levels. Patients suffering from viral infections had PCT levels comparable with those with graft rejection. Hammer *et al*. [58] reported similar findings in a cohort of 78 patients after heart, lung, or heart and lung transplantation. CRP levels were equally elevated in all groups. Hammer *et al*. [64] also reported higher PCT levels in patients with systemic infections than in those with local infection after heart and lung transplantation. PCT levels were almost within normal limits in patients with acute rejection; CRP levels, however, were similarly elevated in all groups [64].

In summary, PCT is useful in differentiating acute graft rejection after heart and/or lung transplantation from bacterial and fungal infections. PCT levels typically remain unchanged after acute rejection but increase markedly after bacterial and fungal infections. Systemic infections are associated with more PCT elevation than is local infection. Viral infections are difficult to identify based on PCT measurements, being only slightly elevated in these patients. CRP levels do not seem to be useful in this setting, because they remain equally elevated regardless of the type of postoperative complication.

## Conclusion

The aims of this qualitative review were to describe the evolution of PCT after cardiac surgery and to assess the value of PCT in terms of diagnosing infection or predicting outcome in these patients. From the available literature, it is difficult to recommend universal cutoff points for PCT which clearly identify and differentiate a normal from a complicated postoperative course. PCT levels should be interpreted, therefore, according to the clinical context. After uncomplicated cardiac surgery, PCT levels increase to achieve a peak level within 24 hours postoperatively and return to normal levels within one week after surgery. The degree of PCT elevation depends on the intraoperative course and the type of the surgical procedure but is unlikely to exceed 5 ng/ml. Patients with a complicated postoperative course, with infection or sepsis syndromes, show higher PCT levels than patients with an uncomplicated course. PCT could be useful in differentiating acute graft rejection of heart and/or lung transplantation from bacterial and fungal, but not from viral, infections.

Concerning the previous limitations and interactions, PCT kinetics seems to be more attractive in identifying patients with infectious complications. There is also evidence that the evolution of PCT levels can be helpful in assessing the adequacy of antibiotic therapy in bacterial infection [12,13].

A meta-analysis by Simon *et al*. [6] showed that the PCT level was more sensitive (88% versus 75%) and more specific (81% versus 67%) than the CRP level in differentiating bacterial from non-infective causes of inflammation. The sensitivity for differentiating bacterial from viral infections was also higher for PCT; the specificities were comparable. PCT also had a higher positive likelihood ratio and lower negative likelihood ratio than did CRP in both groups. The analysis included published studies that evaluated these markers for the diagnosis of bacterial infections in hospitalised patients. In a more recent meta-analysis [10] in adults in ICUs or after surgery or trauma, the summary ROC curve for PCT was better than for CRP. Unfortunately, only a few studies [30,35,39] have reported data on the comparative accuracy between these markers in patients who have undergone cardiac surgery, hindering a meta-analysis of this group. However, the growing body of evidence suggests a minor role for CRP compared with serum PCT in identifying infectious complications in this setting. Further studies are needed to clarify this issue.

## Key messages

• Serum PCT levels typically increase postoperatively after uncomplicated cardiac surgery, reaching a peak level within 24 hours postoperatively.

• The dynamics of PCT levels, rather than absolute values, may be more important for identifying patients with infectious complications after cardiac surgery.

• PCT seems to be superior to CRP in discriminating infection after cardiac surgery.

• PCT levels typically remain unchanged after acute rejection but increase markedly after bacterial and fungal infection.

## Abbreviations

APACHE = acute physiology and chronic health evaluation; CABG = coronary artery bypass graft; CPB = cardiopulmonary bypass; CRP = C-reactive protein; CV = coefficient of variation; ICU = intensive care unit; IL-6 = interleukin-6; MIDCAB = minimally invasive direct coronary artery bypass; MODS = multiple organ dysfunction syndrome; OPCAB = off-pump coronary artery bypass; PCT = procalcitonin; ROC = receiver operating characteristic; SIRS = systemic inflammatory response syndrome; SOFA = sequential organ failure assessment; WBC = white blood cell.

## Competing interests

KR and FB have received fees from BRAHMS AG for speaking and for scientific advice. CS and YS declare that they have no competing interests.

## Authors' contributions

All authors participated in the design of the study. CS and YS contributed to data collection and drafted the manuscript. KR and FB revised the article. All authors read and approved the final manuscript.

## Figures and Tables

**Table 1:** Studies reporting perioperative PCT levels in patients undergoing cardiac surgery

Reference	Year	*n*	Age group	Disease group	PCT assay used and other markers	Results
[66]	1997	48	Adults	Heart transplantation	LUMI-Test	PCT levels were elevated after transplantation and decreased in uncomplicated postoperative course. No PCT elevation was observed with acute graft rejection. Steroid administration in patients with acute rejection had no influence on PCT levels.
[64]^a^	1998	78	Adults	Heart, lung and heart, and lung transplantation	LUMI-Test CRP WBC count	PCT levels were similar between patients with acute graft rejection and non-infected patients. PCT levels were higher in local or systemic infection than rejection. CRP and WBC count were elevated equally in all groups. At discharge, PCT was higher in infected than non-infected patients. At discharge, CRP and WBC count were similar in all groups.
[29]	1998	57	Adults	MIDCAB versus CABG, with uneventful postoperative course	LUMI-Test CRP WBC count	PCT levels were elevated after surgical procedure in both groups and were higher in CABG versus MIDCAB. CRP levels were similar between CABG and MIDCAB. WBC count was elevated postoperatively in CABG versus MIDCAB.
[59]	1998	40	Adults	CABG with ECC ± aprotinin CABG with bacterial infection CABG with SIRS/no infection	LUMI-Test CRP	PCT levels were similar in patients who received aprotinin compared with the control group. PCT levels were less than 0.5 ng/ml at all time points but were higher in bacterial infection versus SIRS. CRP levels were similar in bacterial infection and SIRS.
[35]	1999	59	Adults	CPB; systemic or local infection or control	LUMI-Test CRP	PCT levels increased in all groups, peaked at 24 hours, remained high in patients with systemic infection, and normalised in others. CRP levels increased, peaked at 24 to 48 hours, and remained high in all groups; systemic infection > local infection > control group. PCT levels correlated with CRP levels only in infected patients. PCT: cut-off 4 ng/ml, sensitivity 86%, specificity 98% in predicting infection. CRP: cut-off 180 mg/l, sensitivity 100%, specificity 75% in predicting infection.
[36]	1999	36	Adults	CABG ± CPB; CABG	LUMI-Test CRP	PCT levels increased in the first 4 days, peaked on day 1, and were higher in patients with SIRS than no-SIRS. CRP levels peaked on day 1 and remained high through day 8. After valvular surgery on day 2, CRP levels were similar in patients with SIRS and no-SIRS. No correlation was observed between PCT levels and duration of CPB, aortic clamping, mechanical ventilation, or ICU stay. No correlation was observed between PCT and CRP levels in no-complication group.
[58]^a^	2000	78	Adults	Heart, lung and heart, and lung transplantation	LUMI-Test CRP WBC count	PCT levels were higher in systemic than local infection than rejection than no rejection. PCT levels remained within normal limits in patients with acute graft rejection. CRP levels were equally elevated in all groups. WBC count was similar in all groups.
[37]	2000	74	Adults	CABG/HTx	LUMI-Test CRP WBC count ESR	CABG: PCT levels in sepsis > SIRS > no infection. Patients with no infection had a minimal rise (always less than 0.3 μg/ml) in PCT. Patients with SIRS also had high PCT levels. PCT peaked at 24 hours and normalised in 7 days all patients. HTx: PCT levels were higher in bacterial and fungal infection versus others; CRP was also high in bacterial and fungal infections.
[38]	2000	400	Adults	CPB	LUMI-Test CRP WBC count	WBC count peaked on day 1 in non-infected patients and on day 2 in infected patients (peak 14,000/μl). CRP levels peaked on day 2 in both groups and decreased but did not normalise (infection > no infection). PCT levels peaked on day 1 (infection > no infection) but peaked again on day 6 in infected patients.
[39]	2000	131	Adults	CPB Postoperative infection Septic versus cardiogenic shock	LUMI-Test CRP	PCT levels peaked on day 1, returned to normal values on day 3, and were higher in infected versus non-infected patients. PCT levels correlated to SAPS II score. PCT in patients with septic shock was always greater than 10 ng/ml. CRP levels were high in all patients and did not correlate to PCT. PCT levels were similar in Gram-positive versus Gram-negative infection. PCT levels were higher in septic versus cardiogenic shock. PCT: cut-off 1 ng/ml, sensitivity 85%, specificity 95% in predicting infection. CRP: cut-off 150 mg/l, sensitivity 64%, specificity 84% in predicting infection.
[40]	2000	722	Adults	CPB	LUMI-Test CRP WBC count	PCT levels increased over the first 24 hours; valvular > aortic > CABG. PCT levels were greater in non-survivors versus survivors.
[54]	2000	110	Adults	Cardiac surgery	LUMI-Test CRP WBC count TTR Iron	PCT did not change in patients with uncomplicated postoperative course and was similar in MIDCAB and open surgery. CRP levels peaked on POD 3. CRP levels were similar in minor and major infected patients. WBC count peaked on POD 2. TTR decreased postoperatively, reaching a nadir on PODs 3 and 4. Iron decreased postoperatively, was at its lowest value on POD 2, and was similar in all groups.
[63]	2000	42	Adults	CPB	LUMI-Test Neopterin NO metabolites	PCT levels were higher in complicated than uncomplicated CPB course on PODs 1 and 2. Neopterin was higher in complicated than uncomplicated CPB course. NO metabolites were higher in complicated than uncomplicated CPB course during ECC and on POD 1. PCT cut-off 0.15 ng/ml, positive predictive value 67%, and negative predictive value 82% in predicting postoperative complications.
[65]	2001	110	Adults	Heart, lung, or liver transplantation	LUMI-Test SAA CRP	PCT levels had higher predictive value for bacterial or fungal infection than SAA or CRP. Peak PCT, SAA, and CRP levels were higher in bacterial or fungal infection than viral infection or acute rejection. Peak PCT and SAA levels were slightly higher in patients with viral infection than in those after uneventful course.
[49]	2001	37	Children	Elective repair of congenital heart disease with CPB	LUMI-Test Troponin I (TnI) CK	TnI and CK were higher in cross-clamping time (CCT) greater than 80 minutes versus less than 80 minutes and in ventriculotomy versus atriotomy. PCT levels were higher in CCT greater than 80 minutes versus less than 80 minutes and in ventriculotomy versus atriotomy.
[41]	2001	24	Adults	MODS after CPB	LUMI-Test CRP IL-6 LBP	CRP and LBP levels were similar between study groups irrespective of MODS. IL-6 levels were higher in MODS than SIRS in the first 4 postoperative days. PCT levels were higher in patients with MODS than in those with SIRS. PCT/LPB was higher in patients with MODS with infection than MODS without infection.
[42]	2001	33	Adults	Cardiac surgery and perioperative myocardial infarction (PMI)	LUMI-Test CRP	PCT levels started to rise after CPB, peaked within 24 hours postoperatively, and decreased after 48 hours. CRP levels peaked after 48 hours and remained elevated after 72 hours. PCT levels were higher in PMI versus no PMI postoperatively and correlated to TnI.
[43]	2002	40	Adults	CABG with CPB; dopexamine, epidural anaesthesia, or control	LUMI-Test CRP WBC count TNF Human soluble ICAM-1	PCT/CRP/WBC count was elevated 4 and 18 hours after CPB. PCT levels were lower in patients receiving dopexamine and epidural versus control after 4 and 18 hours. WBC count was lower in dopexamine versus control at 4 hours after CPB. TNF levels were elevated in control 30 minutes after CPB versus baseline and returned to baseline after 18 hours.
[50]	2002	20	Children	Tetralogy of Fallot (TOF)	LUMI-Test CRP IL-6 IL-10	IL-6 levels were elevated in TOF versus healthy infants and preoperatively were higher in TOF versus VSD/AVC. IL-10 levels were lower in TOF versus VSD/AVC preoperatively and during CPB. CRP levels were lower in TOF versus VSD/AVC 24 hours after CPB. PCT levels were elevated after CPB in TOF versus VSD/AVC.
[44]	2002	63	Adults	CABG surgery with CPB with SIRS, severe SIRS, and control	LUMI-Test CRP WBC count	WBC count was similar between sepsis syndromes. CRP levels were higher in SIRS and severe SIRS versus control, with no difference between SIRS and severe SIRS. PCT levels were higher postoperatively in severe SIRS versus SIRS/control, with no difference between SIRS and no SIRS.
[45]	2002	208	Adults	Elective cardiovascular surgery	LUMI-Test CRP Lactate	PCT levels were higher in patients with postoperative complications. PCT, but not CRP, levels correlated with APACHE, SOFA, lactate, duration of ECC, duration of surgery, and ICU stay. PCT: cut-off 2 ng/ml, sensitivity 83.3%, specificity 75.2% in predicting infections.
[67]	2002	40	Adults	ECC + CABG	LUMI-Test WBC count Elastase AT III	PCT levels did not change perioperatively.
[62]^a^	2003	454	Adults	CABG	LUMI-Test Albumin Euroscore COD	In multivariate analysis, serum albumin was associated with poorer outcome than PCT. PCT greater than 2.8 ng/ml discriminated non-survivors.
[55]	2003	28	Adults	CPB	LUMI-Test IL-6, IL-8, IL-18, IL-10, TGF-β	PCT/IL-8/IL-18 levels were higher in non-survivors. (IL-6, IL-10, and TGF-β were not.)
[46]	2003	25	Children	CPB	LUMI-Test CRP IL-6	PCT levels typically peaked at 24 hours and normalised postoperatively after day 5. CRP levels peaked at day 3 and remained elevated. IL-6 peaked at 6 hours and remained elevated. Peak PCT (not CRP/IL-6) levels correlated with duration of CPB, duration of aortic cross-clamping, days of intubation, and ICU days. Only PCT levels were higher in complicated cases.
[47]^b^	2003	5	Adults	Aa disc	LUMI-Test CRP WBC count	PCT levels were higher preoperatively and peaked at 24 hours (likewise CRP). WBC count continued to rise at 48 hours.
[57]	2003	80	Adults	CABG with APACHE II > 20	LUMI-Test	PCT was higher in non-survivors than survivors, in infected than non-infected patients, and in complicated than uncomplicated cases. PCT greater than 5 ng/ml had a sensitivity of 81.5% and a specificity of 45.3% in predicting infection. PCT greater than 10 ng/ml had a sensitivity of 72.2% and a specificity of 51% in discriminating non-survivors.
[68]	2004	63	Adults	OPCAB	LUMI-Test N-BNP	N-BNP/PCT levels were higher in severe SIRS > SIRS > others.
[48]^a^	2004	37	Children	Surgery for congenital heart disease	LUMI-Test IL-6	IL-6 levels increased postoperatively 50-fold independent of CCT, peaked within 24 hours after surgery, and were similar according to CCT, surgical technique, and CBT over the study period. PCT levels postoperatively were higher in CCT greater than 80 minutes versus less than 80 minutes, in ventriculotomy versus atriotomy, and in CBT below 22°C versus above 22°C.
[32]	2004	14	Children	Surgery for congenital heart disease with CPB	LUMI-Test CRP	PCT levels were higher after CPB than preoperative. PCT level peaked on POD 1 and decreased on POD 2. CRP levels were higher after CPB than preoperatively. CRP levels peaked just after CPB and remained high on POD 3.
[33]	2005	32	Adults	Elective CABG	LUMI-Test CRP WBC count	Baseline PCT levels were similar with uncomplicated and complicated postoperative course but peaked at 48 hours in complicated cases, reaching higher levels than uncomplicated cases. CRP/WBC count showed similar kinetics irrespective of the presence of complications.
[34]	2005	108	Adults	Elective thoracic (TC) and cardiac surgery (CABG + CPB/OPCAB)	LUMI-Test IL-6 IL-8 TNF-α CRP LBP IL-2R	IL-6 levels increased postoperatively and were similar in all groups. IL-8 levels increased postoperatively in OPCAB and CABG but not after TC. TNF levels increased postoperatively in OPCAB and TC but not in CABG. CRP and LBP levels increased postoperatively and peaked by the third day. PCT levels peaked after 24 hours and normalised within 5 days but were higher in CABG versus OPCAB. IL-2R levels increased postoperatively and peaked within 3 days.
[31]	2006	53	Children	Elective cardiac surgery ± CPB	LUMI-Test	PCT levels were higher in POD 1 to POD 3 versus baseline. No correlation was observed between PCT levels and bypass duration. In patients with CPB, postoperative PCT values were greater than 1 ng/ml. In patients without CPB, postoperative PCT was less than 1 ng/ml.
[30]	2006	33	Children	Cardiac surgery ± CPB	Kryptor CRP WBC count	PCT levels were higher in SIRS + organ failure than SIRS alone after surgery. PCT levels peaked on POD 1 and decreased until POD 4. CRP levels were similar between SIRS + organ failure and SIRS alone. CRP levels peaked on POD 2. WBC count was similar in SIRS + organ failure and SIRS alone until POD 3, then higher in SIRS + organ failure than SIRS alone. Peak PCT level correlated to ACC, duration of CPB, mechanical ventilation, ICU and hospital stay, mortality, and organ failure development. Peak PCT level of 0.7 ng/ml had a sensitivity of 85% and a specificity of 58% in predicting organ failure. Peak PCT level of 7.7 ng/ml had a sensitivity of 100% and a specificity of 100% in predicting organ failure. Peak PCT level of 5 ng/ml had a sensitivity of 100% and a specificity of 65% in predicting mortality. Peak PCT level of 34.2 ng/ml had a sensitivity of 100% and a specificity of 90% in predicting infection.

^a^Retrospective study; ^b^case report. Aa disc, dissection of the aortic artery; APACHE, acute physiology and chronic health evaluation; AT III, antithrombin III; CABG, coronary artery bypass grafting; CBT, coronary artery bypass time; CK, creatine kinase; COD, colloid osmotic pressure; CPB, cardiopulmonary bypass; CRP, C-reactive protein; ECC, extracorporeal circulation; ESR, erythrocyte sedimentation rate; HTx, heart transplantation; ICAM-1, intercellular adhesion molecule-1; ICU, intensive care unit; IL, interleukin; LPB, lipopolysaccharide binding protein; MIDCAB, minimally invasive coronary artery bypass; MODS, multiorgan dysfunction syndrome; N-BNP, pro-brain natriuretic peptide; NO, nitric oxide; OPCAB, off-pump coronary artery bypass; PCT, procalcitonin; POD, postoperative day; SAA, serum amyloid A; SAPS, simplified acute physiology score; SIRS, systemic inflammatory response syndrome; SOFA, sequential organ failure assessment; TGF-β, transforming growth factor-beta; TNF, tumour necrosis factor; TTR, transthyretin; VSD/AVC, ventricular septal defect/atrioventricular conduit; WBC, white blood cell.
